# Development of an Antifungal and Antimycotoxigenic Device Containing Allyl Isothiocyanate for Silo Fumigation

**DOI:** 10.3390/toxins11030137

**Published:** 2019-03-01

**Authors:** Juan Manuel Quiles, Tiago de Melo Nazareth, Carlos Luz, Fernando Bittencourt Luciano, Jordi Mañes, Giuseppe Meca

**Affiliations:** 1 Laboratory of Food Chemistry and Toxicology, Faculty of Pharmacy, University of Valencia, Av. Vicent Andrés Estellés s/n, 46100 Burjassot, Spain; juan.quiles@uv.es (J.M.Q.); demena@alumni.uv.es (T.d.M.N.); carlos.luz@uv.es (C.L.); jorge.manes@uv.es (J.M.); 2 School of Life Sciences, Pontifícia Universidade Católica do Paraná, Rua Imaculada Conceição 1155, Curitiba 80215-901, Brazil; fernando.luciano@pucpr.br

**Keywords:** *Aspergillus flavus*, *Penicillium verrucosum*, AITC, fungal growth reduction, mycotoxin reduction

## Abstract

The aims of this study were to evaluate the antifungal activity of the bioactive compound allyl isothiocyanate (AITC) against *Aspergillus flavus* (8111 ISPA) aflatoxins (AFs) producer and *Penicillium verrucosum* (D-01847 VTT) ochratoxin A (OTA) producer on corn, barley, and wheat. The experiments were carried out initially in a simulated silo system for laboratory scale composed of glass jars (1 L). Barley and wheat were contaminated with *P. verrucosum* and corn with *A. flavus*. The cereals were treated with a hydroxyethylcellulose gel disk to which 500 µL/L of AITC were added; the silo system was closed and incubated for 30 days at 21 °C. After that, simulated silos of 100 L capacity were used. Barley, wheat, and corn were contaminated under the same conditions as the previous trial and treated with disks with 5 mL of AITC, closed and incubated for 90 days at 21 °C. In both cases, the control test did not receive any antifungal treatment. The growth of the inoculated fungi and the reduction in the formation of AFs and OTA were determined. In the lab scale silo system, complete inhibition of fungal growth at 30 days has been observed. In corn, the reduction of aflatoxin B1 (AFB_1_) was 98.5%. In the 100 L plastic drums, a significant reduction in the growth of *A. flavus* was observed, as well as the OTA formation in wheat (99.5%) and barley (92.0%).

## 1. Introduction

AFs are the foremost harmful category of mycotoxins naturally produced by *Aspergillus* species such as *Aspergillus nomius, Aspergillus flavus*, and *Aspergillus parasiticus* during pre- or postharvest of crops [1,2]. The most important and toxic aflatoxins are the AFB_1_, AFB_2_, AFG_1_, and AFG_2_ [3]. Among these compounds, the AFB_1_ has been classified in Group 1 of the risk of the carcinogen molecules by the International Agency for Research on Cancer [4], and it has been implicated with the development of human hepatic and extra hepatic carcinogenesis [5]. Human exposure to AFs could be due to the intake of contaminated food or by the consumption of milk, meat, and eggs from animals that consumed contaminated feed [6]. The occurrence of AFs in foods from in the Spanish market has been previously reported in several products, such as cereals, pulses, dried fruits and nuts, snacks, breakfast cereals, bread, herbs, or spices [7,8].

OTA is one of the most dangerous mycotoxins and is produced by *Aspergillus* and *Penicillium* species, among which are *Aspergillus ochraceus* [9], *Aspergillus carbonarius* [10], and to a lesser extent *Aspergillus niger* [11] and *Penicillium verrucosum* species [12]. The contamination of food by the presence of OTA is common in Europe. In more than 50% of the 6476 foods analyzed, OTA amounts were detected above the detection limit of 0.01 mg/kg [13]. IARC considers OTA as a possibly carcinogenic compound in humans (Group 2B) [4]. OTA dietary average for citizens of the European Union has been experimentally established in a range of 0.9 (Germany) to 4.6 (Italy) ng/kg. Foods with the greatest OTA contamination are coffee, cereals, spices, and beer [14,15]. When these contaminated foods are ingested, OTA can cause a nephrotoxic, hepatotoxic, and teratogenic effects [16,17].

Methods for controlling mycotoxins are usually preventive, including good agricultural practice and drying of crops after harvest. Some researchers have reported that mycotoxins can be degraded by heat treatment, but the extent of mycotoxin degradation is dependent on temperature, time of exposure, and mainly the contamination level [18]. OTA is a stable molecule, which can resist roasting, brewing, baking, ammoniation, and heat treatment to some extent [19]. Likewise, AFB_1_ seems to be stable up to 150 °C [20]. For this reason, other methods of detoxification have been developed to prevent these mycotoxins in food and feed.

Isothiocyanates (ITCs) are products originated from the enzymatic hydrolysis of glucosinolates, which are sulfur-containing glucosides present in plants of the *Brassicaceae* family. These compounds contribute to the characteristic pungent taste of these vegetables [20] and have been reported as potent antimicrobials [21]. Allyl isothiocyanate (AITC), which is the most studied ITCs, was found to inhibit the growth of yeast, mold, and bacteria at very low levels [22], including molds from the genera *Aspergillus*, *Penicillium*, and *Fusarium* [23,24]. ITCs are characterized by the presence of a –N.C.S group, whose central carbon atom is strongly electrophilic [25]. This electrophilic nature enables ITC to readily bind to thiol and amino groups of amino acids, peptides, and proteins, forming conjugates [21], dithiocarbamate, and thiourea structures [26]. OTA contains a free and readily available amino group and AFs contains a carboxylic group. Therefore, ITCs could be good candidates to react with these mycotoxins.

The objective of this research was to investigate the efficacy of an antifungal device based on the natural compound AITC to reduce the growth of *A. flavus* and *P. verrucosum* in cereals during storage and the mycotoxin production.

## 2. Results

### 2.1. AITC Concentration in Headspace and Cereals in Laboratory Scale Silo

**Figure 1:**
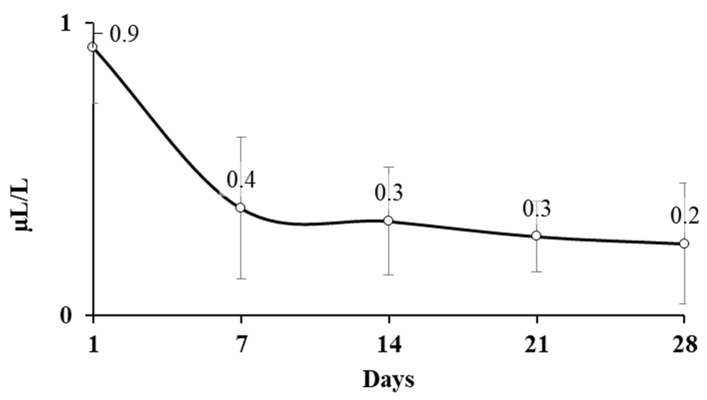
AITC detected in the headspace of the glass jar containing corn, wheat, and barley, used to simulate the storage of the cereals in a lab scale silo system.

Figure 1 shows the AITC present in the headspace of laboratory scale silos. The AITC concentration decreases gradually from 0.92 µL/L at day 1 to 0.25 µL/L at day 30. On the other hand, there was no significant difference in AITC concentration after day 7 up to day 30. These results suggest that either a fraction of AITC left the silo or it was absorbed to grains releasing a constant average concentration ranging from 0.37 to 0.25 µL/L for 30 days.

**Figure 2:**
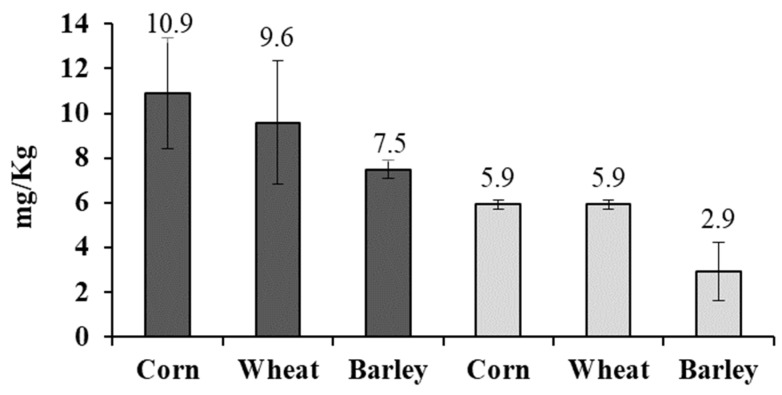
Concentrations of AITC detected in corn, wheat, and barley after 1 (gray) and 30 (white) days of incubation.

The residual concentration of AITC in barley, corn, and wheat was studied on day 1 and 30, and the results are shown in Figure 2. Corn was the most susceptible matrix to AITC penetration, showing 10.9 and 5.9 mg/kg of AITC at days 1 and 30, respectively. Barley grains showed a lower capacity to maintain AITC with 7.5 and 2.9 mg/kg absorbed at days 1 and 30, respectively. Wheat grains did not show a significant difference to barley and corn at day 1, with 9.6 mg/kg of AITC. However, at day 30, wheat grains showed 3 mg/kg more concentration of AITC than barley samples.

### 2.2. Validation Method for the Analysis of Mycotoxins in Cereals

To validate the analytical method, the following parameters such as linearity, recovery, repeatability, reproducibility, limits of detection (LOD) and quantification (LOQ), and the matrix effect for each mycotoxin analyzed were carried out. All the mycotoxins showed good linearity in the working range, with resolution determination coefficients (R2) greater than 0.9922.

Linearity was evaluated using paired matrix calibrations in triplicate at concentrations between 5 and 500 μg/kg. To calculate the matrix effect, the calibration slope from the matrix calibration curve was divided by the slope of the standard calibration curve and multiplied by 100. The value of the recovery was carried out in triplicate for three consecutive days using three addition levels: LOQ, 2 × LOQ, and 10 × LOQ.

The results were between 70.4% and 75.6% and the relative standard deviation (RSD) was less than 17%. The values for intraday repeatability (*n* = 3), expressed as the relative standard deviation of the repeatability (RSDr), varied from 7.5% to 11.6%; and the reproducibility between days (*n* = 5), expressed as the relative standard deviation of the reproducibility (RSDR), varied from 8.2% to 17.3% for the same linearity addition values. LODs and LOQs were calculated by analyzing blank samples enriched with the standard mycotoxins; these parameters have been assessed as the lowest concentration of the molecules studied that showed a chromatographic peak at a signal-to-noise ratio (S/N) of 3 and 10 for LOD and LOQ, respectively (Table 1).

### 2.3. Fungal Growth and Mycotoxin Production in Lab Scale Silo System

**Figure 3:**
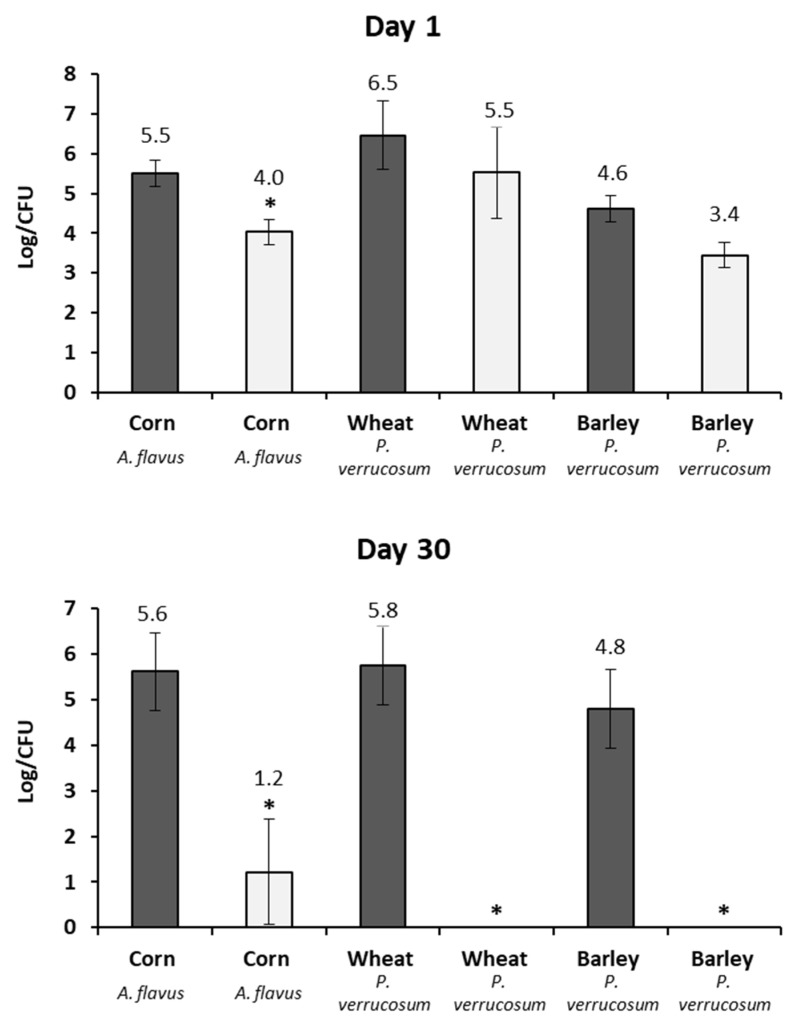
Growth, in lab scale silo system, of the *A. flavus* in corn and of *P. verrucosum* in wheat and barley exposed to the vapor of the AITC after 1 and 30 days of incubation. Samples control (dark gray) and treated samples (clear gray). Significantly different from untreated cereal, *p* ≤ 0.05 (*).

The results of *A. flavus* and *P. verrucosum* growth on barley, corn, and wheat at days 1 and 30 are shown in Figure 3. At day 1, AITC treatment demonstrated a significant reduction in the fungal population of corn, wheat, and barley, reducing in 1.5, 1, and 1.2 log CFU/g, respectively. After 30 days of storage, the fungus growth in the control groups remained stable. However, the treatment reduced the population of fungi in wheat and barley to levels below our limit of detection, while in the corn the reduction was of 4.4 log CFU/g.

**Figure 4:**
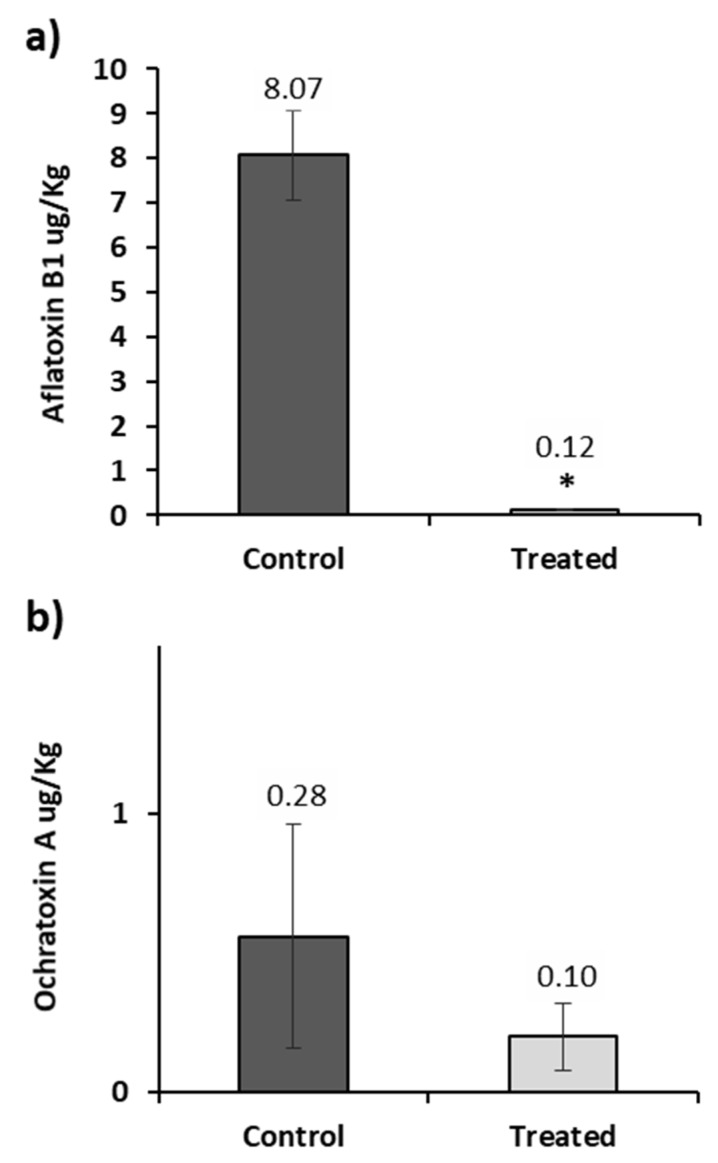
Aflatoxin B1 detected in corn **a**); and ochratoxin A detected in barley **b**) treated with the AITC device in the lab scale silo system at 30 days of incubation. Samples control (dark gray) and treated samples (clear gray). Significantly different from untreated cereal, *p* ≤ 0.05 (*).

In correlation to fungal growth, the mycotoxin production was determined after 30 days of storage and the results are shown in Figure 4. *A. flavus* produced in the control corn 8.07 µg/Kg of AFB_1_ at day 30. This value is above the limit of AFB_1_ in foodstuffs set by the European Commission (EC 165/2010). Therefore, this cereal is classified as inappropriate for human consumption. The AFB_1_ present in the treated corn was 0.12 µg/Kg, representing a reduction of 98.51%. Regarding the values of OTA, the reduction was not significant in barley, whereas in the wheat samples the OTA was not produced even in the control group, reaching values below our limit of detection.

### 2.4. Fungal Growth and Mycotoxin Production in a Small-Scale Silo System

**Figure 5:**
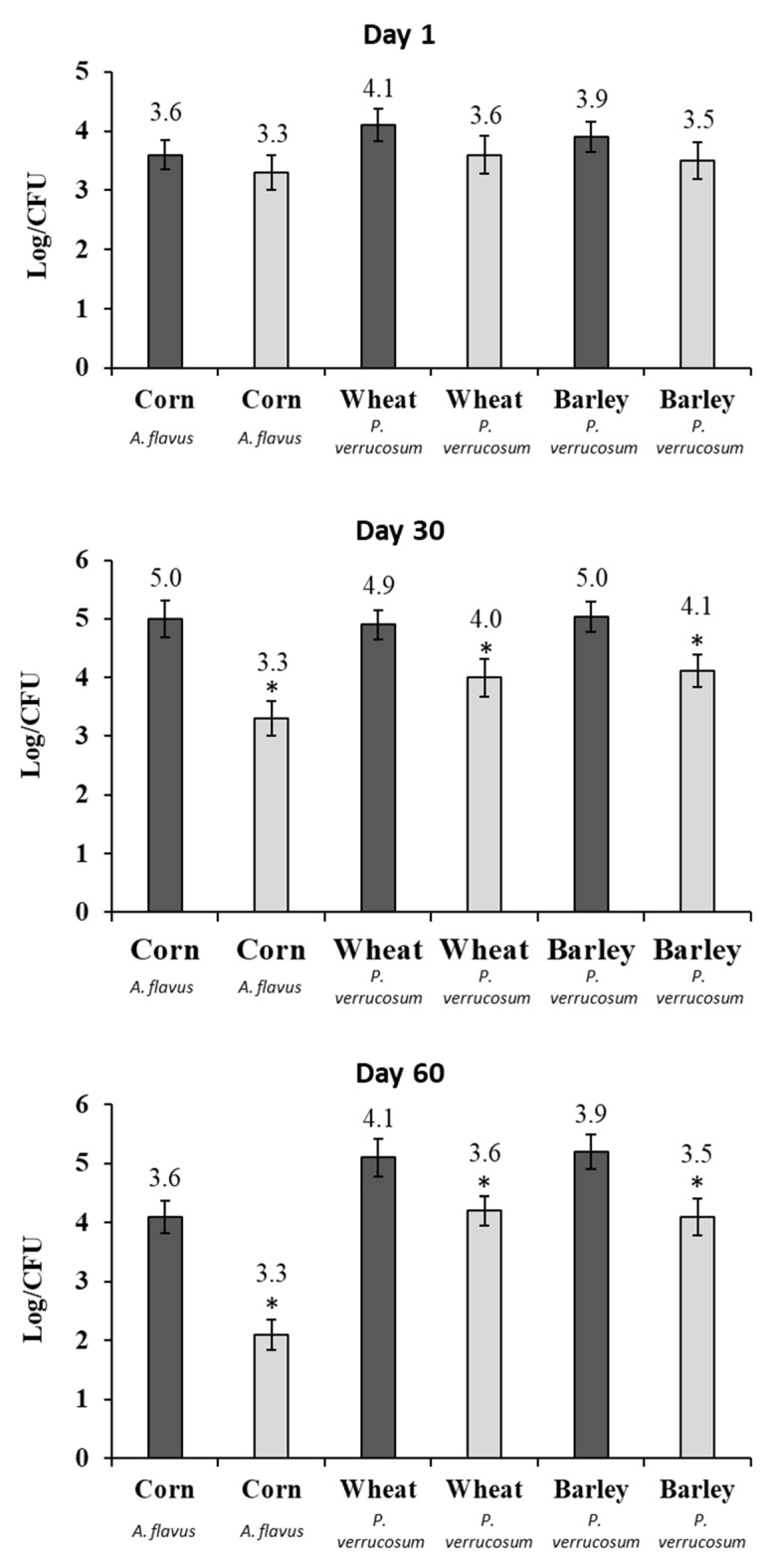
Growth, in small-scale silo system, of the *A. flavus* in corn and of *P. verrucosum* in wheat and barley exposed to the vapor of the AITC after 1, 30, and 60 days of incubation. Control (dark gray) and treated samples (light gray). Significantly different from untreated cereal, *p* ≤ 0.05 (*).

After laboratory scale analysis in silos of 100 L containing 50 Kg of cereal. Barley, corn, and wheat were contaminated and then treated with a gel dispositive developed with 5 mL of AITC. The sampling was realized monthly and the results for microbiological analysis are shown in Figure 5.

At day 1, AITC treatment did not demonstrate a significant reduction in fungal population in corn, wheat, and barley. Similarly, to the laboratory scale silo, after 30 days of storage, the treatment with AITC was able to reduce significantly the fungal growth of *A. flavus* in corn and *P. verrucosum* in barley and wheat. In addition, at the end of the experiment (after 60 days of storage), AITC treatment demonstrated a significant reduction in the fungal population of corn, wheat, and barley, reducing in 2, 0.9, and 1.1 log CFU/g in comparison to the control group, respectively (Figure 5).

**Figure 6:**
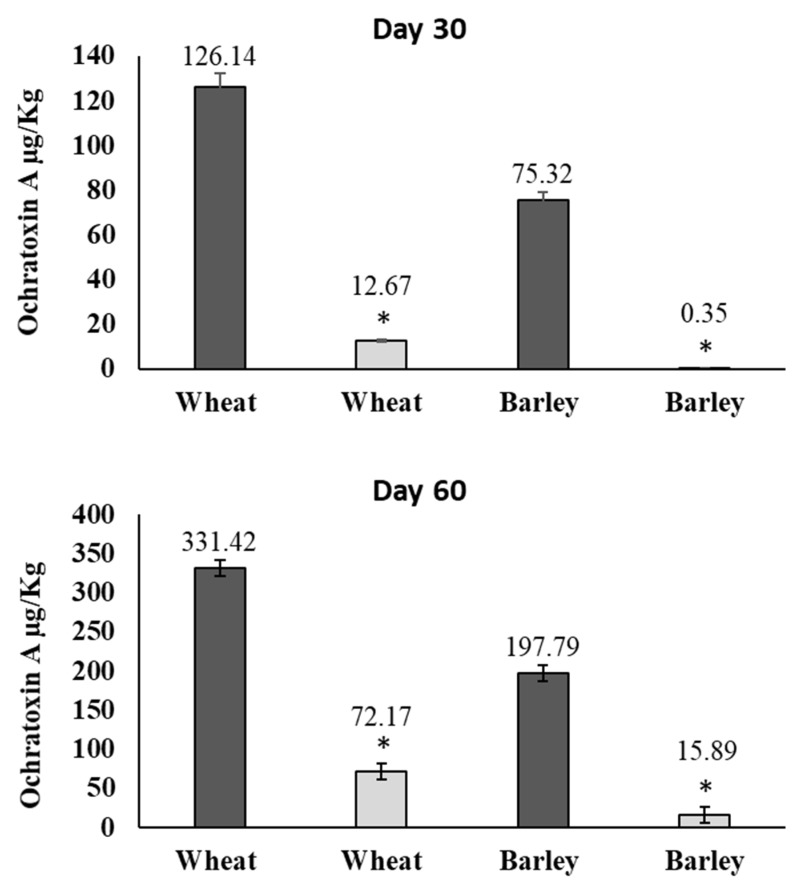
Ochratoxin A detected in wheat and barley treated with the AITC device in the small-scale silo system at 30 and 60 days of incubation. Samples control (dark gray) and treated samples (light gray). Significantly different from untreated cereal, *p* ≤ 0.05 (*).

Along with the fungal growth, the production of mycotoxins was determined at days 30 and 60 of storage (Figure 6). No AFs could be detected in corn contaminated with *A. flavus*. In the samples of wheat and barley contaminated with *P. verrucosum*, the OTA reduction was 90.0% and 99.5% for day 30 and 78.2% and 92.0% for day 60, respectively.

## 3. Discussion

Similarities and differences were identified comparing the results of the lab scale silo system with the results of small-scale silo system. Microbiologically, the exposure to AITC of cereals contaminated with *A. flavus* (corn) and *P. verrucosum* (wheat and barley) reduced, in all experiments, significantly the fungal population after 30 days. However, due to the lower concentration of AITC (50 µL/L) and the micro atmospheres generated by small-scale silo system, the AITC device could not completely inhibit the fungal growth when compared to the lab scale silo system results. In other words, a lower dose of AITC and higher headspace in the small silo system did not reduce the fungal population to the values below to our limit of detection (1.2 logs CFU/g). These results suggest that the effect of AITC is dose depending. In addition, the higher the grain volume, the higher should be the AITC concentration to achieve a total inhibitory effect.

Regarding the production of mycotoxins, differences between the two tests were observed, probably since a moderate inoculum was used to replicate actual contamination conditions of the field. Another difference among the experiments was the reduction of the potential maximum concentration of AITC in the headspace (500 µL/L in the lab silo and 50 µL/L in the small silo) due to issues of scaling and safety of the compound. Even so, in all analysis, a significant reduction in mycotoxin production could be observed among the control and the treated samples when both AFB_1_ and OTA could be produced in matrices.

In the small-scale system, there was an increasing concentration of mycotoxins over time, even in the treated samples. These results could be explained by the presence of the fungal population in the cereals, which allows the mycotoxin production.

In particular, *A. flavus* and *P. verrucosum* depend on oxygen to grow. In our experiment, the headspace in the lab scale silo system and small-scale silo system was around 50% and 20%, respectively. The lower concentration of free oxygen could reduce the regular growth of *A. flavus* and consequently, avoid the AFB_1_ production in the small-scale system. Moreover, the cereals in the small-scale system were not autoclaved, which increased the competitiveness among microorganisms by nutrients.

The application of the AITC to reduce the growth of the fungi mycotoxin producer has been studied previously by other authors. Manyes et al. studied the capacity of AITC produced by the volatilization of a standard solution of the oriental mustard essential oil to prevent the growth of the fungi *A*. *parasiticus* and *Penicillium expansum* [27]. In that study, Petri dishes were inoculated with the mycotoxigenic fungi *A. parasiticus* (producer of AFs) and *P. expansum* (producer of patulin), and the inhibition of micellar growth was observed when they were deposited in the center of the petri dish with 25 µL and 50 µL of AITC, respectively.

Okano et al. assessed the capacity of the AITC obtained by a commercial mustard seed extract to reduce the aflatoxins production by *A. flavus* during the corn storage [28], in a simulated silo condition. The AITC concentration in the headspace of the model system used by the authors reached the highest value of 54.6 μg/L on the day 14 of incubation and remaining stable until 21.8 μg/L until the end of the incubation period. Also, the AITC reduced completely the visible growth of *A. flavus* and the AFs production in both sterilized and unsterilized corn

Delaquis et al. evaluated the capacity of the AITC to reduce the growth of *A. flavus* and *P. expansum* at concentrations of 0.1 μL/L [29]. The experimental model used was the one used in this study. In that case, 2 L flasks were used and inocula of 10^5^ conidia/mL were placed in the presence of different amounts of AITC. These antifungal properties of the AITC were also confirmed by Suhr and Nielsen who inoculated pieces of bread with 10^6^ spores/mL of *Penicillium roqueforti*, *Penicillium corylophilum*, and *A. flavus* and arranged them in closed systems in the presence of mustard essential oil (99% of AITC) [30]. Fungal growth inhibition was observed at concentrations of 1 μL/L. Other studies on the fungicidal activity of AITC against food-disrupting fungi observed the ability of the compound to penetrate the matrix and extend its effect over time. Winther and Nielsen showed that cheeses treated with AITC could absorb this compound, increasing its useful life from 4 to 28 weeks [31].

Quiles et al. developed an active packaging dispositive based on the AITC to reduce the sporulation of *A. parasiticus* and AFs production in fresh pizza doughs during 30 days of inoculation [32]. The antifungal activity of the AITC was compared with untreated samples (fresh pizza doughs without any preservative treatment) and with samples treated with sodium propionate, the classical preservative used in bakery products. After 30 days, the growth of *A. parasiticus* was inhibited with the treatment of AITC at 5 μL/L and 10 μL/L. The reduction of AFs was total at the dose of 10 μL/L.

Nazaret et al. evaluated the capacity of AITC to reduce the production of AFs, beauvericin and enniatin, by *A. parasiticus* and *Fusarium poae* in wheat flour [24]. The analysis of the results showed that the AITC concentration of 0.1 μL/L reduced by 23% the production of the mycotoxins. Also, the application of the AITC at 10 μL/L completely reduced the biosynthesis of the mycotoxins studied during 30 days of incubation.

Tracz et al. evaluated the capacity of AITC at 50, 100, or 500 µL/L to avoid mycotoxin production in corn kernels [33]. Both treatments were able to avoid the production of 12 mycotoxins, including AFB_1_ and Ochratoxin. Saladino et al. analyzed the fungal growth and AFB_1_ reduction by AITC (0.5, 1, or 5 µL/L) in loaf bread [34]. As result, the treatments of 1 and 5 µL/L reduced the AFB_1_ concentration by above 60%. Our results corroborate with these studies, since the AITC at 50 µL/L demonstrated a fungicide and antimicotoxigenic effect, inhibiting the AFB_1_ and OTA synthesis and the fungal growth of *A. parasiticus* and *P.verrucosum* in our small-scale assays.

## 4. Conclusions

The results obtained in this study showed the capacity of the AITC to reduce the growth of the fungi *A. flavus* and *P. verrucosum* in corn, wheat, and barley. The volatilization of the AITC in the headspace of the lab scale silo system was enough to avoid the *A. flavus* and *P. verrucosum* growth in all cereals tested. Moreover, the treatment with AITC device was able to reduce the AFB1 and OTA production in corn and barley, respectively.

In the small-scale silo system, a significant reduction of the *A. flavus* and *P. verrucosum* growth was observed as well as an important reduction of the OTA produced by *P. verrucosum*. The application of the device based on the AITC could be an alternative method to reduce the growth of fungi mycotoxin producer in cereals during the storage phase.

For further studies, the tests carried out in this work will be staggered for 200-ton real silos with naturally contaminated barley and treated with AITC release devices.

## 5. Material and Methods

### 5.1. Chemicals and Microbial Strains

AFB_1_, AFB_2_, OTA (98% purity), AITC, and formic acid (HCOOH) were obtained from Sigma–Aldrich (St. Louis, MO, USA). Methanol and acetonitrile have been obtained by Fisher Scientific (Hudson, NH, USA). Deionized water (<18 MΩ cm resistivity) was produced by a water purification system (Millipore, Bedford, MA, USA). All the chromatographic solvents were filtered through a 0.22 µm membrane filter Scharlau (Barcelona, Spain). Barley, wheat, and corn were provided by Tot Agro (Barcelona, Spain). The peptone water and dextrose potato agar culture medium were obtained from Liofilchem (Teramo, Italy). The strains of *A. flavus* ITEM 8111 were provided by the Microbial Culture Collection of Institute of Sciences and of Food Production (ISPA, Bari, Italy) whereas the *P. verrucosum* VTT D-01847, was obtained from the VTT Culture Collection (Espoo, Finland).

### 5.2. Laboratory Scale Silo System and Antifungal Treatment with the AITC Device

**Figure 7:**
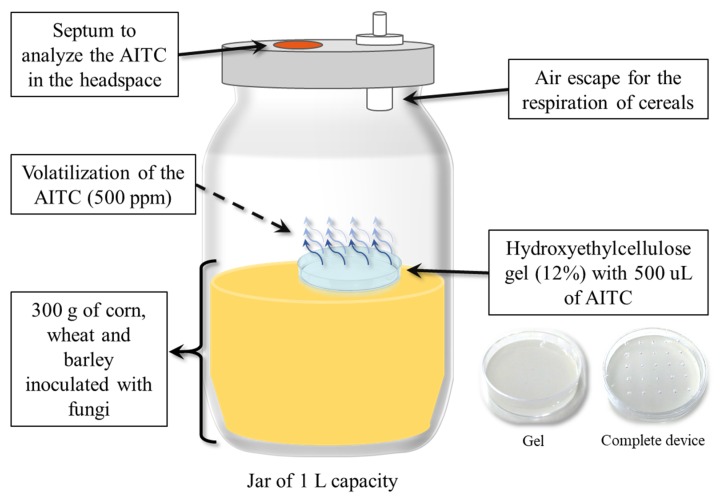
Lab scale silo system used for the treatment of corn, wheat and barley contaminated with *A. flavus* and *P. verrucosum*, and treated with the AITC device.

The silo simulation was carried out as shown in Figure 7. Glass jars of 1 L containing 300 g of cereals were contaminated with 10^4^ conidia/g of *P. verrucosum* (barley and wheat) and *A. flavus* (corn). The cereals were stored for three days to allow fungal adaptation, and treated with 500 µL/L (in the relation of volume of the jar) of AITC into a gel device.

The gel device was manufactured mixing 1.2 g of hydroxyethyl cellulose (gelling agent), 10 mL of water and 500 µL/L of AITC into a Petri dish. The lid of the Petri dish was previously perforated to facilitate the AITC volatilization, as shown in Figure 7. Posteriorly, the antifungal device was placed inside the jars. The jars were closed with adapted lids that contained a septum and an air escape, which allowed AITC analysis in the headspace and cereal respiration, respectively. The samples were stored for 30 days at room temperature. After that, the fungal growth, the mycotoxins contained in the grains, the AITC in the headspace, and the AITC adsorbed by the grains were determined.

### 5.3. AITC Device Application in a Small-Scale Silo System

Fifty kilograms of barley, corn, and wheat were placed inside plastic drums (100 L) separately. Each cereal was contaminated with 10^4^ conidia/g of *P. verrucosum* or *A. flavus*. The barley and wheat were contaminated with *P. verrucosum* and the corn was contaminated with *A. flavus*. Then, the device described in Section 5.2 was adapted to the small-scale silo system.

**Figure 8:**
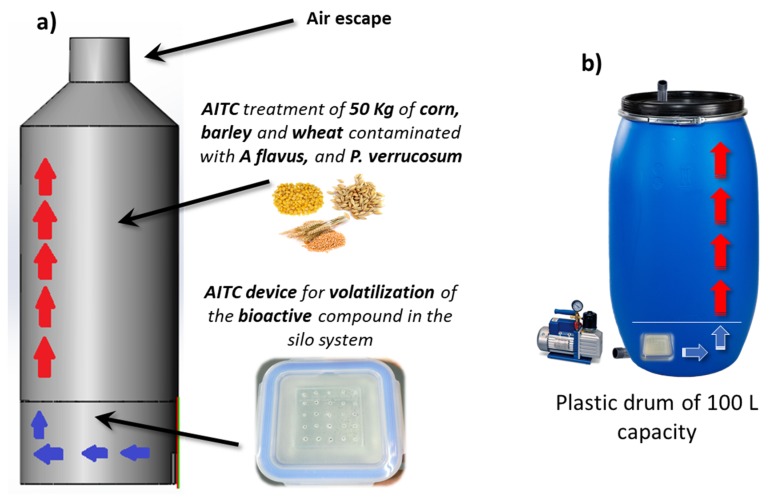
Small-scale silo system used for the treatment of corn, wheat, and barley contaminated with *A. flavus* and *P. verrucosum*, and treated with the AITC device. (**a**) Theoretical silo design; and (**b**) the plastic drum used in this study.

The petri dish was changed for a glass tapper wear, increasing the quantity of the pure AITC contained to 5 mL, in order to obtain 50 µL/L of this bioactive compound in the headspace of the silo. The device was located in the silo bottom and the grains were introduced in the upper part of the silo as shown in Figure 8. A metal grid separated the lower and the upper part of the silo in order to isolate the device and the AITC vapors from the stored cereals. The control group did not receive any antifungal treatment. The analysis carried out on the treated cereals was the same as described in Section 5.2.

### 5.4. Determination of AITC Concentration in the Headspace of the Laboratory Scale Silo

The AITC content in headspace was determined through a septum localized in the lip of a laboratory silo system (Figure 7). The air was recovered using a syringe of 1 mL, and aliquots of 200 µL were injected in a gas chromatograph (GC) with flame ionization detector (FID) (GC 6890, Agilent Technologies Inc., Santa Clara, CA, USA.). The chromatograph was equipped with a 30 × 0.25 mm CP-SIL 88 fused capillary column (Varian, Middelburg, Netherlands). The temperature of the detector arrived at 200 °C with a gradient of temperature that starts at 60 °C. This temperature was maintained for 1 min and increased 8 °C per min up to 100 °C, then maintained for 5 min and finally increased in 15 °C per min up to 200 °C. The gas utilized as the carrier was H_2_ at 5 mL/min. The ionization was realized with H_2_ at 40 mL/min and purified air at 450 mL/min.

### 5.5. Determination of AITC Concentration in the Cereals of the Laboratory Scale Silo

Extraction of AITC from cereals samples was conducted as described by Tracz et al. with some modifications [33]. Five g samples were weighed into 15 mL polyethylene tubes to which 10 mL of methanol was added. The mixture was shaken for 30 min in a water bath (40 °C) and for 10 min in an ultrasonic bath. The samples were centrifuged at 4000 *g* for 5 min at 20 °C. The supernatant (8 mL) was collected and filtered through a 0.22 μm nylon membrane. 20 µL were injected in the LC system, 1220-Infinity (Agilent, Santa Clara, CA, USA) coupled with a diode array detector (LC-DAD) at 236 nm. A Gemini C18 column (Phenomenex, Torrance, CA, USA) 4.6 × 150 mm, 3 μm particle size at 30 °C was used as a stationary phase. The isocratic mobile phase consisted of water/acetonitrile (60:40, *v*/*v*) with a flow rate of 1 mL/min.

### 5.6. Mycotoxin Extraction and LC-MS/MS Analysis of Corn, Barley, and Wheat

The extraction of mycotoxins was carried out following the method described by Serrano et al. with some modifications [35]. Each cereal sample was crushed using a food grinder (Oster Classic Grinder 220e240 V, 50/60 Hz, 600 W, Oster, Valencia, Spain). The resulting particles were mixed, and three 5 g aliquots of each sample were taken in 50 mL plastic falcon tubes. 25 mL of methanol was added to each of these tubes and the samples were homogenized for 3 min by Ultra Ika T18 ultraturrax (Staufen, Germany) at 10,000 rpm. The extract was centrifuged at 4000 rpm during 5 min at 5 °C, and the supernatant was transferred to a plastic flask and evaporated to dryness with a Büchi Rotavapor R-200 (Postfach, Switzerland). The obtained residue was resuspended in 5 mL of methanol, transferred to a 15 mL plastic falcon tube and evaporated with nitrogen gas stream using a multi-sample Turbovap LV evaporator (Zymark, Hopkinton, MA, USA). Finally, the residue was reconstituted in 1 mL of methanol, filtered through a 13 mm/0.22 μm filter and transferred to a 1 mL glass chromatography vial. The liquid-chromatography system consisted of an LC-20AD pump coupled to a 3200QTRAP mass spectrometer (Applied Biosystems, Foster City, CA, USA) using an ESI interface in positive ion mode. The mycotoxins were separated on a Gemini NX C18 column (150 × 2.0 mm I.D, 3.0 mm, Phenomenex, Palo Alto, CA, USA). The mobile phases were the solvent A (5 mM ammonium formate and 0.1% formic acid in water) and solvent B (5 mM ammonium formate and 0.1% formic acid in methanol) at a flow rate of 0.25 mL/min. The elution was carried out using a linear gradient from 0 to 14 min. The injection volume set was of 20 mL, the nebulizer, the auxiliary and the auxiliary gas were set at 55, 50, and 15 psi respectively. The capillary temperature and the ion spray voltage were of 550 °C and 5500 V, respectively. The ions transitions used for the mycotoxin identification and quantification were: *m*/*z* 313.1/241.3 and 284.9 for AFB1, *m*/*z* 315.1/259.0 and 286.9 for AFB2, *m*/*z* 329.0/243.1 and 311.1 for AFG1, *m*/*z* 331.1/313.1 and 245.1 for AFG2, *m*/*z* 404.3/102.1 and 358.1 for OTA.

### 5.7. Determination of the Fungal Population

After the incubation time, 10 g of each sample was transferred to a sterile plastic bag containing 90 mL of sterile peptone water (Oxoid, Madrid, Spain) and homogenized with a stomacher (IUL, Barcelona, Spain) during 30 s. The suspensions formed were serially diluted in sterile plastic tubes containing 0.1% of peptone water. After that, aliquots of 0.1 mL were plated on Petri dishes containing acidified potato dextrose agar (pH 3.5) (Insulab, Valencia, Spain) and the plates were incubated at 25 °C for 7 d before microbial counting. The results were expressed in logs of colony-forming unit/g of cereal (log CFU/g). All analyses were conducted in triplicate.

### 5.8. Statistical Analysis

The Prism version 3.0 software (GraphPad corporation1, La Jolla, CA, USA, 1989) for Windows was used for the statistical analysis of data. The experiments were realized in triplicate and the differences among groups were analyzed by Student’s *t*-test. The level of significance considered was *p* ≤ 0.05.

## Figures and Tables

**Table 1:** LODs, LOQs, recovery, and matrix effect (ME) (%) for AFB1, AFB2, AFG1, AFG2, and OTA in corn, wheat, and barley.

Mycotoxin	LOD (μg/Kg)	LOQ (μg/Kg)	Recovery (%)	ME (%)
AFB1	0.08	0.27	70.4	78.2
AFB2	0.08	0.27	64.2	76.5
AFG1	0.16	0.53	62.8	65.3
AFG2	0.30	1.00	66.1	60.9
OTA	0.05	0.17	75.6	89.7

## References

[1] Majeed S., Iqbal M., Asi M.R., Iqbal S.Z. (2013). Aflatoxins and ochratoxin A contamination in rice, corn and corn products from Punjab, Pakistan. J. Cereal Sci..

[2] Iqbal S.Z., Asi M.R., Ariño A., Akram N., Zuber M. (2012). Aflatoxin contamination in different fractions of rice from Pakistan and estimation of dietary intakes. Mycotoxin Res..

[3] Pittet A. (1998). Natural occurrence of mycotoxins in foods and feeds—An updated review. Rev. Med. Vet..

[4] IARC-International Agency for Research on Cancer (2012). Monographs on the evaluation of carcinogenic risks to humans. A Review of Biological Agents for Human Carcinogens.

[5] Iqbal S.Z., Asi M.R., Ariño A. (2013). Aflatoxins. Brenner’s Encyclopedia of Genetics.

[6] Hammami W., Fiori S., Al Thani R., Ali Kali N., Balmas V., Migheli Q., Jaoua S. (2014). Fungal and aflatoxin contamination of marketed spices. Food Control.

[7] Blesa J., Soriano J.M., Moltó J.C., Mañes J. (2004). Limited survey for the presence of aflatoxins in foods from local markets and supermarkets in Valencia, Spain. Food Addit. Contam..

[8] Van de Perre E., Jacxsens L., Lachat C., El Tahan F., De Meulenaer B. (2015). Impact of maximum levels in European legislation on exposure of mycotoxins in dried products: Case of aflatoxin B1 and ochratoxin A in nuts and dried fruits. Food Chem. Toxicol..

[9] Van der Merwe K.J., Steyn P.S., Fourie L. (1965). Mycotoxins. II. The constitution of ochratoxins A, B, and C, metabolites of *Aspergillus ochraceus* Wilh. J. Chem. Soc. Perkin.

[10] Téren J., Varga J., Hamari Z., Rinyu E., Kevei F. (1996). Immunochemical detection of ochratoxin A in black *Aspergillus* strains. Mycopathologia.

[11] Abarca M.L., Bragulat M.R., Castellá G., Cabañes F.J. (2018). Impact of some environmental factors on growth and ochratoxin A production by *Aspergillus niger* and *Aspergillus welwitschiae*. Int. J. Food Microbiol..

[12] Schmidt-Heydt M., Bode H., Raupp F., Geisen R. (2010). Influence of light on ochratoxin biosynthesis by *Penicillium*. Mycotoxin Res..

[13] Wolff J., Bresch H., Cholmakov-Bodechtel C., Engel G., Garais M., Majerus P., Rosner H., Scheuer R. (2000). Ochratoxin A: Contamination of foods and consumer exposure. Arch. Lebensmittelhyg..

[14] Petzinger E., Weidenbach A. (2002). Mycotoxins in the food chain: The role of ochratoxins. Livestock Prod. Sci..

[15] Rizzo A., Eskola M., Atroshi F. (2002). Ochratoxin a in Cereals, Foodstuffs and Human Plasma. Eur. J. Plant Pathol..

[16] Denli M., Perez J.F. (2010). Ochratoxins in feed, a risk for animal and human health: Control strategies. Toxins.

[17] Reddy L., Bhoola K. (2010). Ochratoxins-food contaminants: Impact on human health. Toxins.

[18] Jackson L.S., Katta S.K., Fingerhut D.D., DeVries J.W., Bullerman L.B. (1997). Effects of baking and frying on the fumonisin B1 content of corn-based foods. J. Agric. Food Chem..

[19] Meca G., Blaiotta G., Ritieni A. (2010). Reduction of ochratoxin A during the fermentation of Italian red wine Moscato. Food Control.

[20] Engel E., Baty C., Le Corre D., Souchon I., Martin N. (2002). Flavor-active compounds potentially implicated in cooked cauliflower acceptance. J. Agric. Food Chem..

[21] Luciano F.B., Holley R.A. (2009). Enzymatic inhibition by allyl isothiocyanate and factors affecting its antimicrobial action against Escherichia coli O157:H7. Int. J. Food Microbiol..

[22] Isshiki K., Tokuoka K., Mori R., Chiba S. (1992). Preliminary examination of allyl-isothiocyanate vapor for food preservation. Biosci. Biotechnol. Biochem..

[23] Manyes L., Luciano F.B., Mañes J., Meca G. (2015). In vitro antifungal activity of allyl isothiocyanate (AITC) against *Aspergillus parasiticus* and *Penicillium expansum* and evaluation of the AITC estimated daily intake. Food Chem. Toxicol..

[24] Nazareth T.M., Bordin K., Manyes L., Meca G., Mañes J., Luciano F.B. (2016). Gaseous allyl isothiocyanate to inhibit the production of aflatoxins, beauvericin and enniatins by *Aspergillus parasiticus* and Fusarium poae in wheat flour. Food Control.

[25] Zhang Y. (2004). Cancer-preventive isothiocyanates: Measurement of human exposure and mechanism of action. Mutat. Res..

[26] Cejpek K., Valusek J., Velísek J. (2000). Reactions of allyl isothiocyanate with alanine, glycine, and several peptides in model systems. J. Agric. Food Chem..

[27] Okano K., Ose A., Takai M., Kaneko M., Nishioka C., Ohzu Y., Odano M., Sekiyama Y., Mizukami Y., Nakamura N. (2015). Inhibition of aflatoxin production and fungal growth on stored corn by allyl isothiocyanate vapor. J. Food Hyg. Soc. Jpn..

[28] Delaquis P.J., Sholberg P.L. (1997). Antimicrobial activity of gaseous allyl isothiocyanate. J. Food Prot..

[29] Suhr K.I., Nielsen P.V. (2003). Antifungal activity of essential oils evaluated by two different application techniques against rye bread spoilage fungi. J. Appl. Microbiol..

[30] Winther M., Nielsen P.V. (2006). Active packaging of cheese with allyl isothiocyanate, an alternative to modified atmosphere packaging. J. Food Prot..

[31] Quiles J.M., Manyes L., Luciano F., Manes J., Meca G. (2015). Antimicrobial compound allylisothiocyanate against the *Aspergillus parasiticus* growth and its aflatoxins production in pizza crust. Food Chem. Toxicol..

[32] Tracz B.L., Bordina K., Nazareth T.M., Costa L.B., Macedo R.E.F., Meca G., Luciano F.B. (2017). Assessment of allyl isothiocyanate as a fumigant to avoid mycotoxin production during corn storage. LWT.

[33] Saladino F., Quiles J.M., Luciano F.B., Mañes J., Fernandez-Franzón M., Meca G. (2017). Shelf life improvement of the loaf bread using allyl, phenyl and benzyl isothiocyanates against *Aspergillus parasiticus*. LWT.

[34] Serrano A.B., Font G., Mañes J., Ferrer E. (2013). Emerging Fusarium mycotoxins in organic and conventional pasta collected in Spain. Food Chem. Toxicol..

[35] Pitt J.I., Hocking A.D. (1997). Fungi and FoodSpoilage.

